# Heterogeneous leukocyte telomere trajectories and inflammatory resolution 12 months after mild COVID-19: an exploratory cohort study

**DOI:** 10.3389/fragi.2026.1866981

**Published:** 2026-06-30

**Authors:** Daiane Renata dos Santos, Daniela Valadão Freitas Rosa, Arnaldo Santos Leite, Carolina Coimbra Marinho, Maria Aparecida Camargos Bicalho, Marco Aurelio Romano-Silva, Débora Marques de Miranda, Alexandre Guimarães de Almeida Barros

**Affiliations:** Medical School, Universidade Federal de Minas Gerais, Belo Horizonte, Brazil

**Keywords:** cellular senescence, hepatocyte growth factor, inflammaging, leukocyte telomere length, multiplex cytokines, SARS-CoV-2

## Abstract

**Background:**

The longitudinal interplay between leukocyte telomere length (LTL) and persistent immune activation after mild SARS-CoV-2 infection remains incompletely characterized.

**Methods:**

In this exploratory observational study, 51 adults (76.5% female; mean age 39.0 ± 9.7 years) with RT-PCR-confirmed mild COVID-19 had blood sampled in the acute symptomatic phase and at 12 months. Relative LTL was quantified by qPCR (Cawthon T/S method, 36B4 reference). A 45-analyte multiplex bead-based immunoassay quantified circulating cytokines, chemokines and growth factors. Paired changes were assessed with Wilcoxon signed-rank tests; cross-sectional and longitudinal associations between immune mediators and LTL were evaluated by Spearman correlation with Benjamini–Hochberg false discovery rate (FDR) control and by multivariable linear regression adjusted for baseline LTL, age, sex and comorbidity burden.

**Results:**

Forty-eight participants had paired LTL data. Group-level T/S ratio increased modestly between the acute phase and 12 months (mean Δ = +0.0094; Wilcoxon p = 0.041; Cohen’s d_z = 0.31), but interindividual trajectories were markedly heterogeneous (52% increased, 33% decreased, 15% unchanged). Twenty of 45 cytokines decreased significantly between timepoints (FDR <0.05), consistent with systemic resolution of acute inflammation. Among residual immune mediators at 12 months, hepatocyte growth factor (HGF) was inversely associated with the LTL trajectory (Spearman ρ = −0.48; p = 0.0005; FDR = 0.024). This association persisted in a multivariable model adjusted for baseline LTL, age, sex and comorbidity count (β = −0.040; 95% CI −0.061 to −0.018; p = 0.0006), and the model explained 43% of variance in T/S_1_. Cytokines previously highlighted in similar cohorts (IL-7, IL-9, IL-17A, EGF) showed univariable correlations that did not survive FDR correction.

**Conclusion:**

Twelve months after mild SARS-CoV-2 infection, leukocyte telomere trajectories are highly individual, while most acute-phase inflammatory mediators have resolved. Residual circulating HGF, a pleiotropic factor recognized as a component of the senescence-associated secretory phenotype but also involved in tissue repair, endothelial activation, and metabolic signaling, was the only mediator robustly associated with the longitudinal LTL trajectory after multiple-testing correction and may identify a subgroup with persistent tissue-remodeling or senescence-associated activity. This association is interpreted as exploratory and hypothesis-generating rather than as a validated biomarker. Given the absence of an uninfected control group, modest sample size, and qPCR-based LTL quantification, these findings should be interpreted as hypothesis-generating.

## 1 Introduction

Beyond its acute respiratory manifestations, SARS-CoV-2 infection has emerged as a model human stressor for studying the molecular and immunological consequences of acute viral disease, including possible long-term effects on biological aging (Nalbandian et al., 2021; Davis et al., 2023; Franceschi et al., 2018; del Rio et al., 2020). Telomeres, the nucleoprotein structures protecting chromosomal termini, shorten with each somatic cell division and integrate the cumulative impact of genetic background, oxidative stress, and inflammatory exposure (Blackburn et al., 2015; Aviv and Shay, 2018). Leukocyte telomere length (LTL), the most widely studied surrogate of replicative cellular age, is therefore plausibly responsive to systemic immune activation, including that triggered by acute viral infection (Ilmonen et al., 2008; Bellon and Nicot, 2017).

Several cross-sectional studies have documented associations between shorter LTL and severe COVID-19 outcomes, often interpreted as reflecting either pre-existing biological vulnerability or acute infection-related telomeric stress (Wang et al., 2021; Sanchez-Vazquez et al., 2021). However, longitudinal evidence on whether and how LTL evolves after recovery from SARS-CoV-2 infection remains scarce, particularly in non-hospitalized patients (Mongelli et al., 2021; Su et al., 2022; Cao et al., 2022). Two key open questions are whether LTL trajectories are uniform or heterogeneous across recovered individuals, and whether persistent immune activation contributes to interindividual variation in telomeric remodeling (Schneider et al., 2022; Arbeev et al., 2020).

In parallel, multiplex profiling of circulating cytokines, chemokines and growth factors has shown that acute SARS-CoV-2 infection induces a broad inflammatory signature that resolves only partially in some individuals, a phenomenon implicated in post-acute sequelae of COVID-19 (Su et al., 2022; Lucas et al., 2020; Phetsouphanh et al., 2022). Whether residual immune mediators at the post-acute stage are coupled to LTL trajectories is poorly characterized.

We therefore conducted an exploratory longitudinal observational analysis of a Brazilian cohort of adults with mild COVID-19, aiming to describe paired changes in LTL and in 45 circulating immune mediators between the acute phase and 12 months after infection, and to identify, through FDR-corrected analyses, immune markers whose levels at 12 months are associated with the magnitude and direction of LTL change. We do not test a single causal hypothesis, rather, we aim to generate hypotheses about candidate post-viral biomarkers of cellular aging that warrant validation in larger, controlled cohorts.

## 2 Methods

### 2.1 Study design and ethics

This exploratory longitudinal observational study was nested within a prospective cohort of adults with confirmed SARS-CoV-2 infection enrolled at the Medical School of the Universidade Federal de Minas Gerais (UFMG), Belo Horizonte, Brazil. The study protocol was approved by the UFMG Research Ethics Committee (CAAE 37688020.1.0000.5149) and conducted in accordance with the Declaration of Helsinki. All participants provided written informed consent at enrollment and at follow-up. Reporting follows the STROBE statement (von Elm et al., 2007).

### 2.2 Participants

**FIGURE 1:**
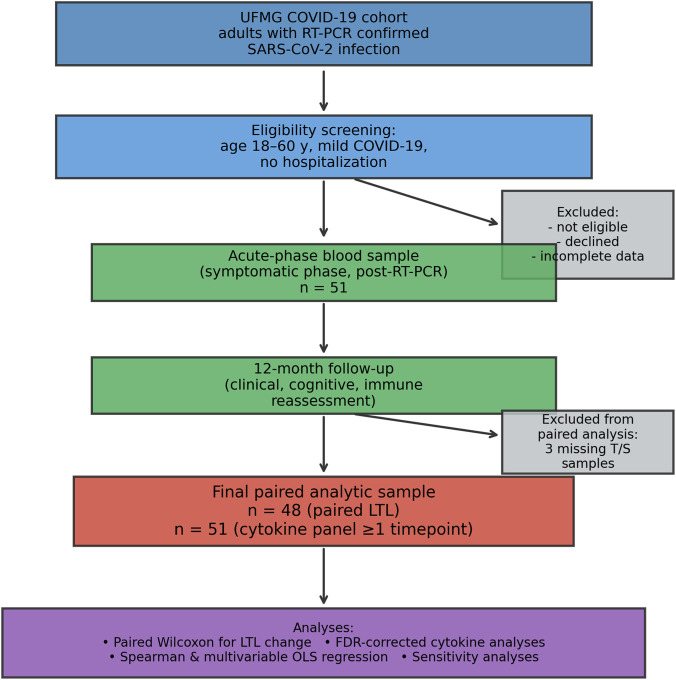
Study flowchart and analytic sample. From the UFMG COVID-19 longitudinal cohort, 51 adults with mild COVID-19 had acute-phase blood sampling; 48 had paired LTL data at 12 months. Cytokine analyses included 49–51 participants depending on analyte completeness.

Inclusion criteria were age 18–60 years, RT-PCR–confirmed SARS-CoV-2 infection, predominantly mild COVID-19 not requiring hospitalization or supplemental ventilation, and availability of paired biological material for at least one of the analytic platforms. Exclusion criteria were age outside the 18–60 range, hospitalization due to COVID-19, and pre-existing major neurological or psychiatric disorders. Baseline blood sampling occurred during the acute symptomatic phase, after diagnostic confirmation; follow-up sampling occurred 12 months later as part of a comprehensive reassessment that included clinical evaluation. Of 51 participants enrolled with acute-phase data, 48 had paired LTL data and were included in the primary longitudinal telomere analyses (Figure 1).

### 2.3 Telomere length quantification

Genomic DNA was extracted from peripheral blood leukocytes by high-salt precipitation (Lahiri and Schnabel, 1993). Relative LTL was measured as the telomere-to-single-copy gene (T/S) ratio by quantitative real-time PCR following Cawthon’s monochromatic method (Cawthon, 2002), using primers Tel-1 (5′-GGTTTTTGAGGGTGAGGGTGAGGGTGAGGGTGAGGGT-3′)/Tel-2 (5′-TCC​CGA​CTA​TCC​CTA​TCC​CTA​TCC​CTA​TCC​CTA​TCC​CTA-3′) for telomeres and 36B4-u (5′-CAGCAAGTGGGAAGGTGTAATCC-3′)/36B4-d (5′-CCC​ATT​CTA​TCA​TCA​ACG​GGT​ACA​A-3′) for the acidic ribosomal phosphoprotein P0 (36B4) reference gene. All reactions were run in triplicate across two independent runs; a reference DNA pool served as inter-run calibrator. Results are presented as relative T/S ratios.

DNA purity and concentration were verified by UV spectrophotometry (NanoDrop) with acceptance criteria A260/280 ≈ 1.8–2.0. Samples were normalized to a working concentration of 0.47 ng/μL (75 ng diluted in a 160-µL final volume), and 20 µL of this working stock was added per reaction, yielding approximately 9.4 ng of template DNA per well. Each qPCR reaction had a final volume of 50 µL and contained 30 µL of an in-house SYBR Green-based master mix at the following final concentrations: 1× reaction buffer, 2 mM MgCl_2_, 200 µM dNTPs, 0.2× SYBR Green, 50 nM ROX as passive reference, and 0.25 µL of Platinum Taq DNA polymerase per reaction. For the telomere assay, final primer concentrations were 270 nM Tel-1 (forward) and 900 nM Tel-2 (reverse), following the asymmetric design of Cawthon (2002); for the 36B4 assay, 300 nM 36B4-u (forward) and 500 nM 36B4-d (reverse). Amplification was performed on an Applied Biosystems 7500 Real-Time PCR System (Thermo Fisher Scientific) with the following cycling protocol: an initial holding stage of 50 °C for 2 min and 95 °C for 10 min, followed by 30 cycles of 95 °C for 15 s and 54 °C for 2 min for the telomere assay, and 35 cycles of 95 °C for 15 s and 58 °C for 2 min for the 36B4 assay; melting-curve analysis at the end of each run confirmed amplicon specificity. The two assays were run on separate plates with identical sample layouts: both timepoints from a given participant were placed on the same plate (rows A–C for the 12 month sample, rows D–F for the acute-phase sample), each in triplicate, in order to minimize plate-specific bias on the within-participant paired comparison. Amplification efficiencies were determined from a serial dilution curve and met standard criteria (90%–110% efficiency, *R* ^2^ ≥ 0.98). No-template, no-primer and no-Taq negative controls were included on every plate and consistently failed to amplify. Per-sample triplicate Ct values were inspected for technical quality; the median ΔCt SD (Ct[Tel] − Ct[36B4], computed within each well-pair across the three replicates) was 0.22 cycles, with 68% of samples falling within the Cawthon technical tolerance of <0.3 cycles, corresponding to a propagated technical coefficient of variation at the T/S level of approximately 15%. Samples whose triplicate ΔCt SD exceeded 0.5 cycles (12% of paired observations) were re-inspected, and where the deviation could be attributed to a single outlier well, that well was excluded prior to averaging.

### 2.4 Multiplex cytokine profiling

Plasma was obtained from EDTA-anticoagulated blood by centrifugation at 1,500 × g for 10 min at 4 °C, aliquoted, and stored at −80 °C. Forty-five circulating immune mediators were quantified by Luminex xMAP-based multiplex bead-based immunoassay (Bio-Rad), with concentrations derived from five-parameter logistic standard curves. Limits of detection ranged from 0.5 to 10 pg/mL, depending on the analyte. The full panel included: pro-inflammatory cytokines (IL-1α, IL-1β, IL-2, IL-6, IL-12p70, IL-15, IL-17A, IL-18, IL-22, IL-23, IL-27, TNF-α, TNF-β, IFN-α, IFN-γ, GM-CSF, LIF), Th2/regulatory cytokines (IL-4, IL-5, IL-9, IL-10, IL-13, IL-31), homeostatic and lymphopoietic factors (IL-7, IL-21, IL-1RA, SCF), chemokines (IL-8/CXCL8, IP-10/CXCL10, MCP-1/CCL2, RANTES/CCL5, MIP-1α, MIP-1β, Eotaxin, GRO-α/CXCL1, SDF-1α), and growth/neurotrophic factors (EGF, FGF-2, HGF, NGF-β, BDNF, PDGF-BB, VEGF-A, VEGF-D, PlGF-1).

### 2.5 Statistical analysis

Continuous variables are reported as mean ± SD or median (IQR), depending on the results of the Shapiro-Wilk normality test. Paired changes between timepoints were tested with the Wilcoxon signed-rank test. Cross-sectional associations between immune mediators and LTL were assessed with Spearman’s rank correlation. Given the 45-analyte panel, all univariable associations were corrected for multiple testing using the Benjamini–Hochberg false discovery rate (FDR), with FDR <0.05 considered statistically significant and FDR <0.10 considered suggestive.

Determinants of relative LTL at 12 months (T/S_1_) and of the longitudinal change in LTL (Δ = T/S_1_ − T/S_0_) were further explored with multivariable linear regression. A core-adjusted model included baseline T/S_0_ (only for the T/S_1_ outcome), age, sex, and comorbidity count (sum of self-reported diabetes, asthma, hypothyroidism, dyslipidemia, neurological disease, stroke, and cardiovascular history). Cytokines that survived FDR-corrected univariable screening were added to the extended models after log_10_ transformation. Regression assumptions were checked through residual inspection. A sensitivity analysis excluded participants with extreme |Δ(T/S)| > 0.05. Two-sided p-values <0.05 were considered statistically significant; analyses were performed in Python 3.11 (NumPy, SciPy 1.11, pandas 2.2).

For the paired LTL analysis (n = 48), the study had approximately 80% power to detect a true effect size of Cohen’s d_z = 0.40 at two-sided α = 0.05, and approximately 60% power for the observed effect size (d_z = 0.31); for Spearman rank correlations against Δ(T/S) at n = 48, the study had approximately 80% power to detect a true |ρ| = 0.40, but only approximately 40%–70% power for smaller effect sizes (|ρ| = 0.25–0.35). Negative findings are therefore interpreted as not statistically detectable in this sample rather than as evidence of the absence of association. Given the exploratory scope of the study, FDR correction was applied to the primary panel of 45 univariable cytokine–LTL associations; secondary analyses (subgroup stratifications, sensitivity exclusions, and *post hoc* descriptive comparisons) were not subjected to additional multiple testing correction and should be regarded as descriptive.

### 2.6 Variables collected and not collected during follow-up

For each participant, the protocol systematically recorded age, biological sex, and a structured self-reported comorbidity questionnaire (diabetes mellitus, asthma, hypothyroidism, dyslipidemia, neurological disease, stroke history, and prior cardiovascular events). The interval between RT-PCR confirmation and acute-phase blood sampling was not uniformly recorded. Vaccination status against SARS-CoV-2 during the 12-month follow-up was not systematically captured at the time of the original protocol, and reinfection events were not systematically captured. Long-COVID symptoms were assessed at the 12-month visit using a structured clinical interview administered by the study team; the present analysis does not include these symptom data. The SARS-CoV-2 variant was not determined; the cohort spans 2020–2021, a period of mixed circulation of ancestral SARS-CoV-2 lineages and the Gamma and Delta variants in Brazil. The following variables were not collected and are acknowledged as limitations: body mass index, smoking status (current/former/never; pack-years), alcohol use, regular medication use (including immunomodulators, statins, antihypertensives, hormone replacement, and contraceptives), ethnicity, leukocyte differential counts at either timepoint, and intercurrent non-COVID infections during follow-up. Because of these gaps, multivariable models could not be adjusted for several established or plausible LTL and cytokine determinants; this is addressed explicitly in the Limitations.

## 3 Results

### 3.1 Cohort characteristics

Of 51 participants with acute-phase data, 48 had paired LTL measurements, and 49–51 had cytokine data at one or both time points, depending on the analyte (Figure 1). The cohort was predominantly female (39/51, 76.5%), with a mean age of 39.0 ± 9.7 years (range, 23–59 years). At least one self-reported chronic condition was present in 28 (54.9%) participants, most commonly dyslipidemia (23.5%), self-reported cardiovascular history (15.7%), asthma (13.7%), and hypothyroidism (11.8%) (Table 1).

**TABLE 1:** Demographic and clinical characteristics of the analytic cohort.

Variable	Female (n = 39)	Male (n = 12)	Total (n = 51)
Age, years (mean ± SD)	40.3 ± 9.6	35.0 ± 9.2	39.0 ± 9.7
Dyslipidemia, n (%)	9 (23.1%)	3 (25.0%)	12 (23.5%)
Cardiovascular history*, n (%)	6 (15.4%)	2 (16.7%)	8 (15.7%)
Asthma, n (%)	6 (15.4%)	1 (8.3%)	7 (13.7%)
Hypothyroidism, n (%)	5 (12.8%)	1 (8.3%)	6 (11.8%)
Diabetes mellitus, n (%)	1 (2.6%)	0 (0%)	1 (2.0%)
Stroke history, n (%)	1 (2.6%)	0 (0%)	1 (2.0%)
Neurological disease, n (%)	2 (5.1%)	0 (0%)	2 (3.9%)
≥1 comorbidity, n (%)	22 (56.4%)	6 (50.0%)	28 (54.9%)

* Self-reported cardiovascular history; not necessarily angiographically confirmed coronary artery disease.

### 3.2 Longitudinal changes in leukocyte telomere length

**FIGURE 2:**
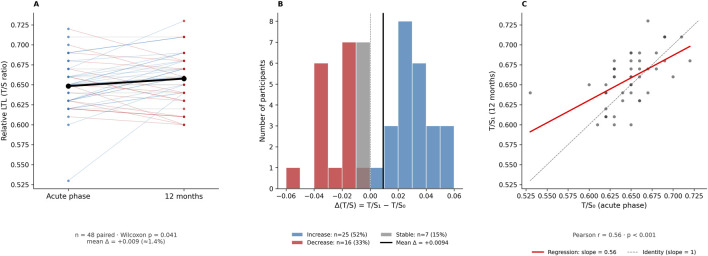
Longitudinal changes in relative leukocyte telomere length (LTL). **(A)** Paired T/S ratios from the acute phase to 12 months in n = 48 participants; group-level mean indicated by red squares. **(B)** Distribution of Δ(T/S); the mean shift is small relative to between-individual spread. **(C)** Scatter of T/S_1_ *versus* T/S_0_, indicating moderate tracking and regression to the mean.

Mean relative LTL increased modestly between the acute phase (T/S_0_ = 0.648 ± 0.032) and 12 months (T/S_1_ = 0.658 ± 0.032), with a mean paired Δ of +0.0094 (95% CI 0.0008 to 0.0179; Wilcoxon p = 0.041; Cohen’s d_z = 0.31; Figure 2A). The magnitude of this group-level shift (≈1.4%) falls within the expected range of qPCR T/S technical variability (see Methods, Section 2.3) and should not be interpreted as biologically meaningful telomere lengthening or “recovery”. However, this group-level signal masked substantial interindividual heterogeneity: 25 participants (52.1%) showed an increase in T/S, 16 (33.3%) a decrease, and 7 (14.6%) no measurable change (Figure 2B). T/S_0_ and T/S_1_ were positively correlated (Pearson r = 0.56) but with clear regression to the mean (Figure 2C), and a sensitivity analysis excluding participants with |Δ(T/S)| > 0.05 attenuated the group-level effect (Wilcoxon p = 0.12), indicating that the modest mean change at the cohort level is driven by a subgroup with pronounced longitudinal variation rather than by a uniform cohort-wide trend. We therefore frame the primary LTL finding as heterogeneity of individual trajectories around a near-stable group mean.

### 3.3 Systemic resolution of acute-phase immune mediators

**FIGURE 3:**
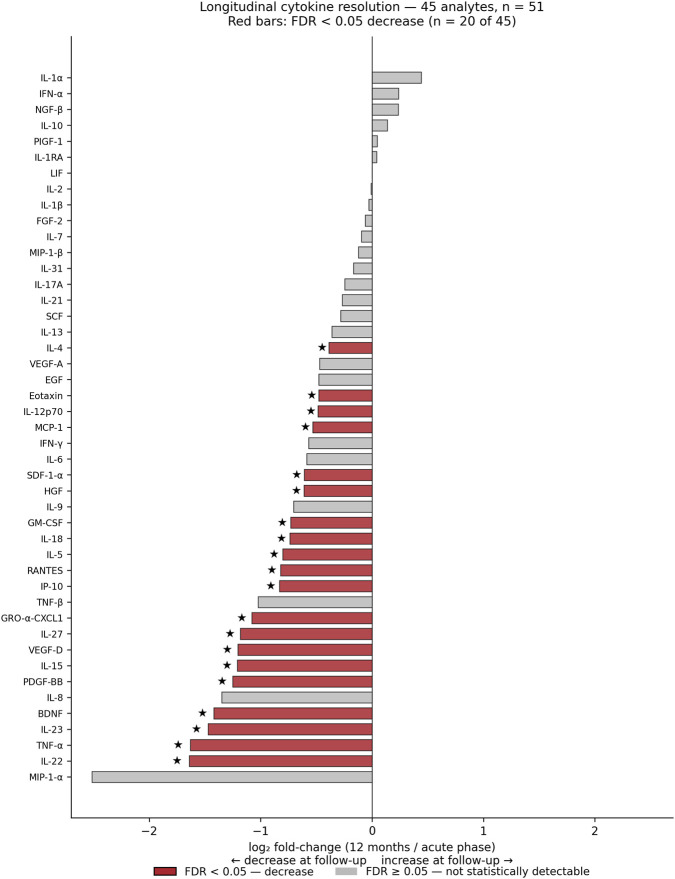
Magnitude of paired decrease for the 20 circulating immune mediators with statistically significant longitudinal change after FDR correction. Percentages indicate change in median concentration (12 months relative to acute phase).

Of the 45 circulating immune mediators, 20 showed statistically significant decreases from the acute phase to 12 months after FDR correction (FDR <0.05; Figure 3). Strongest reductions were observed for IL-22 (−68%, FDR <0.001), TNF-α (−68%, FDR <0.001), IL-23 (−64%, FDR <0.001), BDNF (−63%, FDR <0.001), PDGF-BB (−58%, FDR <0.001), VEGF-D (−57%, FDR <0.001), IL-15 (−57%, FDR <0.01), IL-27 (−56%, FDR <0.001), GRO-α/CXCL1 (−53%, FDR <0.001), and RANTES (−43%, FDR <0.001). No cytokine increased significantly between timepoints. This pattern is consistent with broad systemic resolution of acute viral inflammation, with several mediators (notably HGF, EGF, IL-7) remaining detectable at 12 months and providing a substrate for cross-sectional associations with LTL.

### 3.4 Hepatocyte growth factor at 12 months is associated with telomere trajectory

**FIGURE 4:**
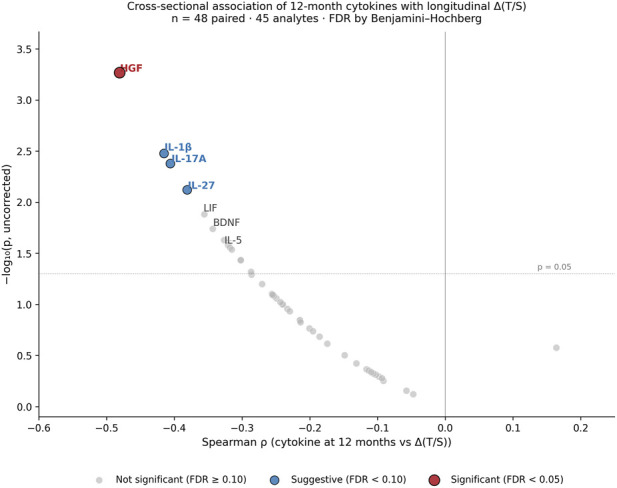
Volcano plot of the cross-sectional associations between the 45 circulating immune mediators quantified at 12 months and the longitudinal change in leukocyte telomere length [Δ(T/S)] (n = 48 paired samples). Each point denotes a single analyte; the abscissa represents the Spearman rank correlation coefficient (ρ) and the ordinate the ‐log_10_ of the uncorrected p‐value. Multiple comparisons were controlled via the Benjamini‐Hochberg false discovery rate (FDR). Points are color‐coded by significance tier: red, FDR < 0.05 (HGF); blue, FDR < 0.10 (suggestive: IL‐1β, IL‐17A, IL‐27); grey, FDR ≥ 0.10 (non‐significant). The dashed horizontal line indicates the uncorrected p = 0.05 threshold. HGF was the sole mediator exhibiting a statistically significant inverse association with Δ(T/S) after FDR correction.

To identify residual immune mediators associated with LTL, we conducted FDR-corrected Spearman analyses pairing each of 45 cytokines at 12 months with both T/S_1_ and Δ(T/S) (Figure 4). For T/S_1_, no cytokine survived FDR correction; previously reported associations with EGF, IL-7, IL-9, and IL-17A had uncorrected p ≈ 0.014–0.038 but FDR ≥0.42 (Supplementary Table S1).

**FIGURE 5:**
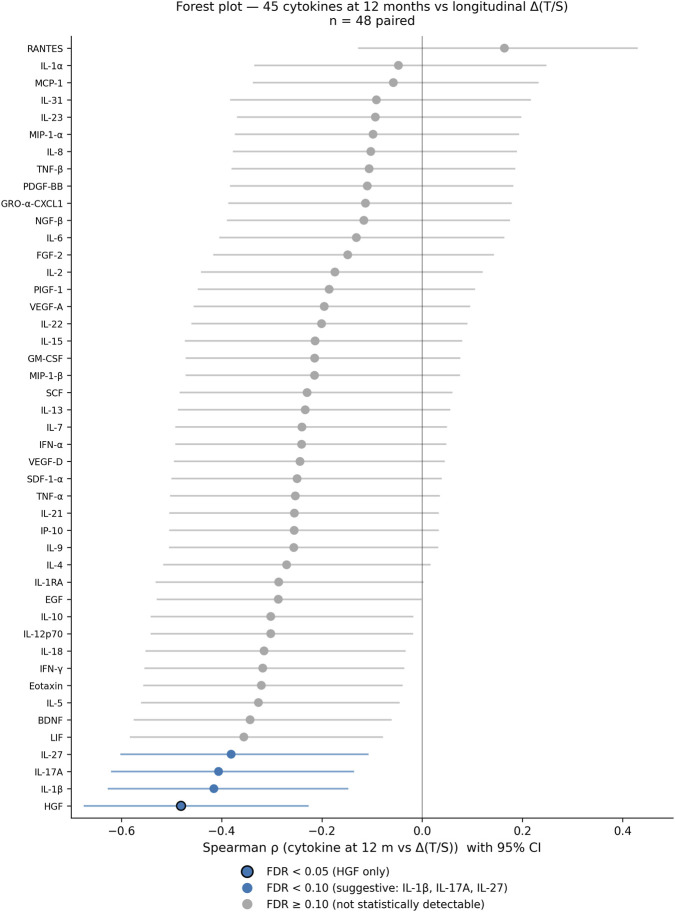
Forest plot of Spearman correlations (with 95% bootstrap CIs) between cytokines at 12 months and the 12-month LTL change. Only HGF survives FDR correction at the <0.05 level; IL-1β, IL-17A, and IL-27 are FDR-suggestive (FDR <0.10).

**FIGURE 6:**
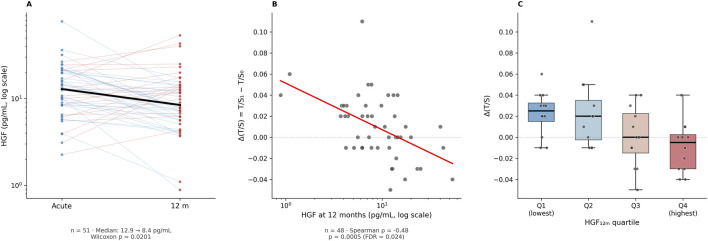
Hepatocyte growth factor (HGF) and leukocyte telomere length. **(A)** Paired plasma HGF concentrations (log scale) from the acute phase to 12 months (n = 51), showing a significant longitudinal decline (median 12.9 to 8.4 pg/mL; Wilcoxon p = 0.0201). **(B)** Cross-sectional association between log_10_(HGF) at 12 months and the longitudinal LTL change Δ(T/S) (n = 48 paired). **(C)** Δ(T/S) stratified by HGF_12m_ quartile, showing a graded inverse pattern.

By contrast, the longitudinal LTL change [Δ(T/S)] was significantly associated with HGF at 12 months (Spearman ρ = −0.48; p = 0.0005; FDR = 0.024; Figure 5), with directionally consistent but FDR-borderline signals for IL-1β, IL-17A, and IL-27 (uncorrected p < 0.01; FDR 0.06–0.09). Stratification by HGF_12m_ quartile revealed a graded relationship: participants in the lowest HGF quartile had a mean Δ(T/S) of +0.022 (telomere lengthening), while those in the highest quartile had a mean Δ(T/S) of −0.009 (telomere shortening) (Kruskal–Wallis p = 0.021; Figure 6C).

### 3.5 Multivariable models

In a multivariable linear regression of T/S_1_ on baseline T/S_0_, age, sex, and comorbidity count (n = 48; adjusted *R* ^2^ = 0.26), only baseline T/S_0_ was independently associated with follow-up LTL (β = +0.547, p < 0.001). Adding log_10_(HGF) at 12 months substantially improved fit (adjusted *R* ^2^ = 0.43) with a higher HGF inversely and independently associated with T/S_1_ (β = −0.040, 95% CI −0.061 to −0.018; p = 0.0006), while T/S_0_ remained the dominant determinant (β = +0.603, p < 0.001) and age, sex, and comorbidity count remained non-significant (Table 2).

**TABLE 2:** Multivariable linear regression model for relative LTL at 12 months (T/S_1_).

Predictor	β	SE	95% CI	p-value
Intercept	0.317	0.078	0.160 to 0.475	0.0002
**Baseline T/S**_**0**_	**0.603**	0.114	0.372 to 0.833	**<0.001**
Age (years)	−0.0002	0.0004	−0.001 to 0.001	0.578
Sex (female = 1)	−0.006	0.009	−0.024 to 0.012	0.491
Comorbidity count	−0.0001	0.005	−0.010 to 0.010	0.992
**log**_**10**_ **(HGF**_**12m**_ **)**	**−0.040**	0.011	−0.061 to −0.018	**0.0006**

OLS, regression; n = 48; adjusted *R* ^2^ = 0.43; F-test p < 0.001. Bold values indicate statistical significance (p < 0.05).

## 4 Discussion

In this exploratory observational analysis of adults with mild COVID-19, three observations emerge from a paired molecular dataset spanning the acute phase and 12 months after infection. First, leukocyte telomere length showed a small mean increase at the group level but markedly heterogeneous individual trajectories, with roughly equal proportions of participants gaining or losing measurable telomeric content. Second, the dominant longitudinal signal in circulating immunology was the broad resolution of acute inflammatory mediators: 20 of 45 cytokines and growth factors decreased significantly after FDR correction. Third, residual hepatocyte growth factor (HGF) at 12 months was the only immune mediator robustly and independently associated with the LTL trajectory, surviving multiple-testing correction and adjustment for baseline LTL, age, sex, and comorbidity count.

The observation of heterogeneous telomere trajectories rather than uniform attrition or recovery is consistent with the view that LTL responds to acute inflammatory stress in an individual-specific manner, modulated by baseline biological reserves, immune cell turnover, and compensatory regulatory pathways (Aviv and Shay, 2018; Arbeev et al., 2020; López-Otín et al., 2023). The modest mean increase observed here is small relative to assay-level variability, and a sensitivity analysis confirms that the group-level signal depends on a subgroup with substantial change. Such within-individual heterogeneity also helps reconcile previously reported cross-sectional associations between shorter LTL and adverse COVID-19 outcomes (Wang et al., 2021; Sanchez-Vazquez et al., 2021) with the absence of a uniform, infection-related shortening of LTL over time in mild disease.

The association between residual circulating HGF and LTL trajectory is biologically plausible but should be interpreted with caution given HGF’s pleiotropy. HGF is an established component of the senescence-associated secretory phenotype (SASP), released by senescent cells and tissues undergoing chronic regenerative stress, and is implicated in inflammaging and post-injury remodeling (Coppé et al., 2010; Igarashi et al., 2022). It is also a tissue-repair mitogen and is influenced by endothelial activation, hepatic and metabolic status, and a range of chronic inflammatory states unrelated to telomere biology; we therefore avoid framing HGF as a direct molecular link between immune signaling and telomere remodeling. Its persistence at higher concentrations 12 months after a mild viral infection plausibly indexes residual cellular stress and senescence burden, which in turn may track with replicative pressure and DNA damage signaling at telomeres (Nassour et al., 2024). A more balanced interpretation is that elevated residual HGF at 12 months identifies a subgroup with persistent tissue-remodeling or senescence-associated inflammatory activity and a less favorable LTL trajectory, without implying a specific causal pathway. Even if validated in larger cohorts, the cross-sectional HGF_12m_–Δ(T/S) association is consistent with at least three causal structures that cannot be discriminated from the present design: (i) elevated HGF directly drives telomere stress via SASP-mediated signaling at telomeres; (ii) pre-existing senescent or short-telomere cells secrete more HGF (reverse causation), with HGF as a downstream marker of cells already committed to senescence; and (iii) an unmeasured exposure (residual viral reservoir, low-grade systemic inflammation, or BMI/lifestyle factors not captured in our protocol) drives both elevated HGF and altered telomere dynamics. These three structures are illustrated in Supplementary Figure S1, and the present observational design cannot adjudicate between them. Suggestive secondary signals from IL-1β, IL-17A, and IL-27, although not reaching FDR significance, are directionally consistent with persistent low-grade innate and Th17 activation (Korn et al., 2009; Garlanda et al., 2013) but should be regarded as exploratory and in need of independent validation.

Importantly, several cytokine–LTL associations highlighted in earlier descriptive analyses of similar COVID-19 cohorts (notably IL-7, IL-9, IL-17A, and EGF) did not survive FDR correction in our data. We interpret these uncorrected signals as consistent with chance findings given multiple testing rather than as reproducible biological associations. This caveat aligns with broader concerns about over-interpretation of multiplex cytokine panels in modestly powered studies and underscores the need for prespecified analytic strategies and multiple-testing control (Benjamini and Hochberg, 1995).

A specific note on causal interpretation is warranted, given the study design. Because no uninfected control group was sampled and no pre-infection baseline LTL is available, the heterogeneous telomere trajectories described here cannot be causally attributed to SARS-CoV-2 infection. Within-individual differences in T/S over 12 months may reflect normal biological variation in LTL, which is influenced by hematopoietic clone dynamics, intercurrent infections, lifestyle exposures, hormonal status, assay variability, regression-to-the-mean effects, or post-viral processes, and the current design cannot disentangle these. Over-attributing such changes to mild COVID-19 carries concrete risks: it can inflate the perceived biological impact of mild infection on cellular aging, distort the prior probability assigned to post-viral aging hypotheses, misdirect future mechanistic work toward effects that may be of modest magnitude or non-existent, and propagate uncertain premises into downstream studies that build on this literature. We therefore deliberately frame all longitudinal LTL observations as descriptive and the HGF–Δ(T/S) association as exploratory and hypothesis-generating, rather than as evidence of a virus-induced cellular-aging phenotype. Controlled designs with matched uninfected comparators, ideally with pre-pandemic LTL measurements and orthogonal LTL technologies (such as TRF Southern blot or single-telomere length analysis), will be required to test whether mild SARS-CoV-2 infection itself meaningfully contributes to telomere variability beyond the technical and biological background.

Limitations should temper interpretation. First, the absence of an uninfected control group and pre-infection baseline precludes causal attribution of LTL changes to SARS-CoV-2 itself. Second, the qPCR-based T/S method has well-documented technical variability; for our assay, the median within-sample ΔCt SD across triplicate well-pairs was 0.22 cycles, corresponding to a propagated technical coefficient of variation in T/S of approximately 15%. This technical floor is roughly an order of magnitude larger than the observed group-level mean change in T/S (+1.4%), which constrains inferences about cohort-level mean LTL trends but does not undermine the within-individual rank-based associations captured in the regression analyses. Third, the modest sample size limits statistical power, especially for multivariable models, and stratified subgroup analyses must be considered exploratory. Fourth, restriction to mild COVID-19 narrows generalizability to moderate or severe disease phenotypes. Fifth, several established or plausible determinants of LTL and circulating immune mediators were not systematically captured and could not enter the multivariable models: specifically, body mass index, smoking status (current/former/never and pack-years), alcohol use, regular medication use (notably immunomodulators, statins, antihypertensives and hormonal therapy), ethnicity, SARS-CoV-2 vaccination status during the 12-month interval, documented reinfection events, intercurrent non-COVID infections, and self-reported long-COVID symptoms. The interval from RT-PCR confirmation to acute-phase blood sampling was also not uniformly documented, which may have introduced within-cohort variability in acute-phase cytokine reference values. Cellular composition correction (lymphocyte, monocyte, and granulocyte fractions) was not feasible from the available samples, which limits inferences about subset-driven LTL variability in particular. These gaps may bias both telomeric and cytokine estimates; therefore, we explicitly avoid causal language for any specific exposure. Sixth, the predominantly female composition of the cohort (76.5%) and the absence of viral variant data (the cohort spans different pandemic waves, with likely heterogeneity in circulating SARS-CoV-2 lineages) limit the generalizability of any subgroup inferences. Together, these constraints define the analyses presented here as descriptive and hypothesis-generating; we discourage extrapolation beyond the design’s boundaries.

In conclusion, these data depict a post-acute COVID-19 landscape in which most acute-phase inflammatory mediators have resolved by 12 months, while individual leukocyte telomere trajectories vary substantially. Among residual immune mediators, hepatocyte growth factor, a pleiotropic mediator of tissue repair, endothelial activation, and senescence-associated signaling, emerged as the only analyte robustly associated with the LTL trajectory after multiple-testing correction. We interpret this association as exploratory and hypothesis-generating: it may identify a subgroup with persistent tissue remodeling or senescence-associated inflammatory activity, rather than directly linking immune signaling to telomere remodeling. Validation in larger, controlled cohorts with matched uninfected comparators, orthogonal LTL measurement techniques, and mechanistically oriented endpoints is required before HGF can be considered a candidate biomarker of post-viral cellular aging.

## Data Availability

The raw data supporting the conclusions of this article will be made available by the authors, without undue reservation.
