# Supplementary figures and images for: Heterogeneous leukocyte telomere trajectories and inflammatory resolution 12 months after mild COVID-19: an exploratory cohort study
Source: Front Aging. 2026 Jun 30;7:1866981. doi: 10.3389/fragi.2026.1866981 (PMC13367134; doi:10.3389/fragi.2026.1866981)

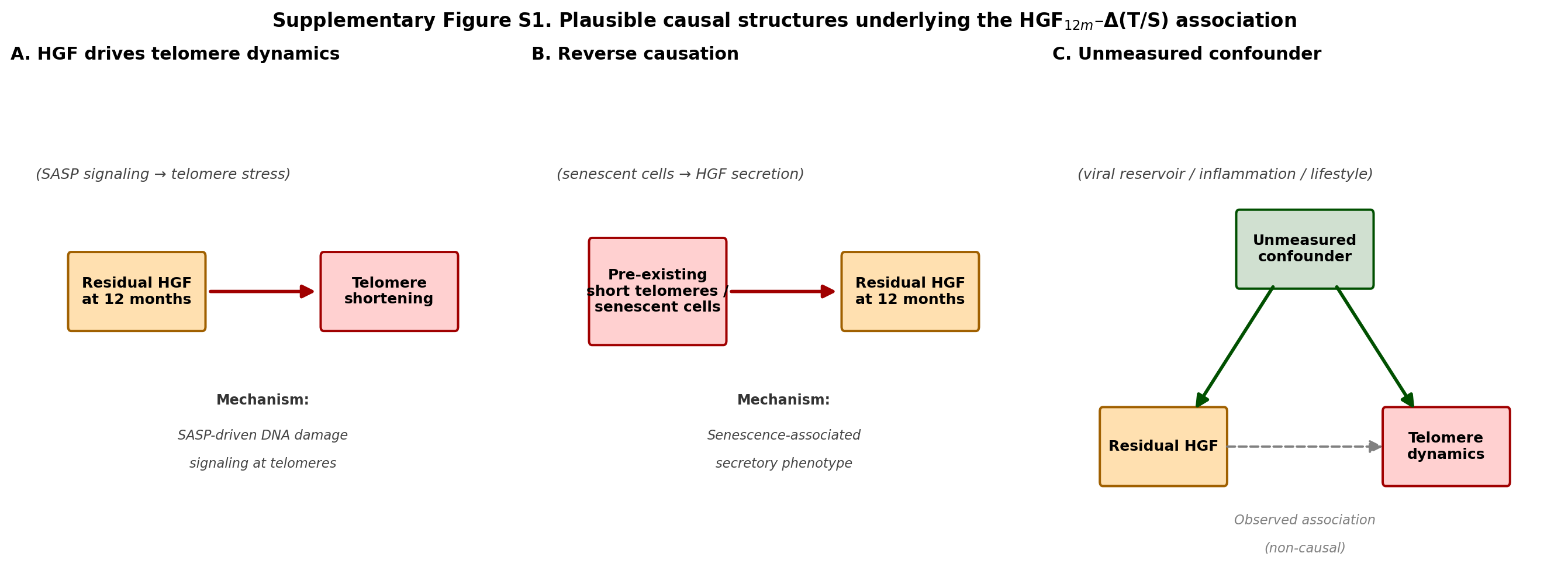

Supplement: Supplementary file 1 [file Image1.tiff]
