# Supplementary material for: Heterogeneous leukocyte telomere trajectories and inflammatory resolution 12 months after mild COVID-19: an exploratory cohort study
Source: Front Aging. 2026 Jun 30;7:1866981. doi: 10.3389/fragi.2026.1866981 (PMC13367134; doi:10.3389/fragi.2026.1866981)
Supplement: Supplementary file 2 [file DataSheet1.pdf]

Supplementary Material

Heterogeneous trajectories of leukocyte telomere length and systemic resolution of inflammatory mediators 12 months after mild SARS-CoV-2 infection

Supplementary Table S1. Cross-sectional Spearman correlations between circulating immune mediators at 12 months and relative leukocyte telomere length at 12 months (T/S_1), with Benjamini-Hochberg FDR correction across the 45-analyte panel.

Cytokine	n	Spearman_rho	p_uncorrected	FDR_BH
EGF	49	-0.3503	0.0136	0.4227
IL-9	49	-0.3230	0.0236	0.4227
IL-7	49	-0.3023	0.0348	0.4227
IL-17A	49	-0.2974	0.0380	0.4227
IL-15	48	-0.2679	0.0657	0.4227
TNF-alpha	49	-0.2636	0.0673	0.4227
IL-23	49	-0.2628	0.0682	0.4227
NGF-beta	49	-0.2517	0.0810	0.4227
HGF	49	-0.2441	0.0909	0.4227
IL-8	49	-0.2386	0.0988	0.4227
IL-4	49	-0.2275	0.1159	0.4227
RANTES	49	0.2228	0.1238	0.4227
IL-31	44	-0.2271	0.1383	0.4227
GM-CSF	49	-0.2117	0.1442	0.4227
IFN-gamma	49	-0.2108	0.1459	0.4227
IL-27	49	-0.2078	0.1520	0.4227
LIF	49	-0.2033	0.1612	0.4227
IL-2	48	-0.2018	0.1691	0.4227
IL-6	48	-0.1833	0.2124	0.5031
TNF-beta	49	-0.1703	0.2420	0.5313
IL-10	49	-0.1661	0.2539	0.5313
IL-13	49	-0.1595	0.2736	0.5313
GRO-alpha-CXCL1	49	-0.1567	0.2821	0.5313
IFN-alpha	49	-0.1563	0.2834	0.5313
IL-1-beta	49	-0.1518	0.2979	0.5362
IL-5	49	-0.1462	0.3160	0.5470
IL-22	49	-0.1423	0.3294	0.5490
BDNF	48	-0.1298	0.3792	0.6095
MIP-1-beta	49	0.1192	0.4148	0.6436
PIGF-1	49	-0.1154	0.4299	0.6449
IL-18	49	-0.1036	0.4785	0.6946
MIP-1-alpha	49	-0.0854	0.5598	0.7872
VEGF-D	49	-0.0784	0.5922	0.8076
IL-12p70	49	-0.0685	0.6402	0.8274
SCF	49	-0.0643	0.6605	0.8274
SDF-1-alpha	49	0.0641	0.6619	0.8274

Eotaxin	49	-0.0524	0.7205	0.8691
IL-1RA	48	0.0504	0.7339	0.8691
VEGF-A	49	-0.0343	0.8149	0.9403
IP-10	49	-0.0270	0.8537	0.9605
IL-21	49	-0.0226	0.8774	0.9630
FGF-2	49	-0.0066	0.9639	0.9721
PDGF-BB	49	-0.0065	0.9647	0.9721
MCP-1	49	-0.0056	0.9696	0.9721
IL-1-alpha	47	0.0052	0.9721	0.9721

No analytes survived FDR correction at the 0.05 threshold.

Supplementary Table S2. Spearman correlations between circulating immune mediators at 12 months and the longitudinal change in relative leukocyte telomere length [$\Delta(T/S) = T/S_1 - T/S_0$], with Benjamini-Hochberg FDR correction.

Cytokine	n	Spearman_rho	p_uncorrected	FDR_BH
HGF	48	-0.4810	5.00e-4	0.0243
IL-1-beta	48	-0.4154	0.0033	0.0627
IL-17A	48	-0.4061	0.0042	0.0627
IL-27	48	-0.3810	0.0075	0.0849
LIF	48	-0.3557	0.0131	0.1179
BDNF	47	-0.3432	0.0182	0.1304
IL-5	48	-0.3266	0.0235	0.1304
Eotaxin	48	-0.3208	0.0262	0.1304
IFN-gamma	48	-0.3180	0.0276	0.1304
IL-18	48	-0.3154	0.0290	0.1304
IL-12p70	48	-0.3021	0.0369	0.1384
IL-10	48	-0.3021	0.0369	0.1384
EGF	48	-0.2870	0.0479	0.1643
IL-1RA	47	-0.2862	0.0511	0.1643
IL-4	48	-0.2704	0.0630	0.1891
IL-9	48	-0.2561	0.0789	0.1961
IP-10	48	-0.2553	0.0799	0.1961
IL-21	48	-0.2549	0.0804	0.1961
TNF-alpha	48	-0.2530	0.0828	0.1961
SDF-1-alpha	48	-0.2495	0.0873	0.1964
VEGF-D	48	-0.2437	0.0951	0.1969
IFN-alpha	48	-0.2405	0.0997	0.1969
IL-7	48	-0.2398	0.1006	0.1969
IL-13	48	-0.2332	0.1106	0.2074
SCF	48	-0.2295	0.1166	0.2098
MIP-1-beta	48	-0.2147	0.1428	0.2387
GM-CSF	48	-0.2145	0.1432	0.2387
IL-15	47	-0.2136	0.1494	0.2401
IL-22	48	-0.2007	0.1714	0.2659

VEGF-A	48	-0.1954	0.1833	0.2750
PIGF-1	48	-0.1856	0.2067	0.3000
IL-2	47	-0.1741	0.2418	0.3400
RANTES	48	0.1639	0.2655	0.3621
FGF-2	48	-0.1486	0.3135	0.4149
IL-6	47	-0.1313	0.3789	0.4871
NGF-beta	48	-0.1164	0.4308	0.5385
GRO-alpha-CXCL1	48	-0.1132	0.4437	0.5396
PDGF-BB	48	-0.1096	0.4584	0.5429
TNF-beta	48	-0.1059	0.4737	0.5466
IL-8	48	-0.1025	0.4881	0.5491
MIP-1-alpha	48	-0.0980	0.5074	0.5569
IL-23	48	-0.0935	0.5275	0.5652
IL-31	43	-0.0913	0.5602	0.5863
MCP-1	48	-0.0575	0.6978	0.7136
IL-1-alpha	46	-0.0474	0.7545	0.7545

HGF was the only analyte to survive FDR correction at the 0.05 threshold (FDR = 0.024). IL-1β, IL-17A, and IL-27 had FDR-suggestive associations (FDR < 0.10).

Supplementary Table S3. Paired longitudinal change in 45 circulating immune mediators between the acute phase and 12 months (Wilcoxon signed-rank test) with Benjamini-Hochberg FDR correction.

Cytokine	n_paired	Median_acute	Median_12mo	log2FC	p_uncorrected	FDR_BH
IL-22	46	15.328	4.915	-1.641	0.0000	0.0000
RANTES	51	18.485	10.460	-0.8215	0.0000	0.0000
IL-23	51	73.140	26.375	-1.472	0.0000	1.00e-4
TNF-alpha	51	13.190	4.260	-1.631	0.0000	1.00e-4
IL-27	51	56.100	24.710	-1.183	0.0000	3.00e-4
VEGF-D	51	3.400	1.475	-1.205	1.00e-4	4.00e-4
BDNF	47	2.060	0.7700	-1.420	1.00e-4	4.00e-4
PDGF-BB	51	20.750	8.720	-1.251	1.00e-4	4.00e-4
GRO-alpha-CXCL1	49	3.425	1.620	-1.080	1.00e-4	6.00e-4
Eotaxin	51	1.685	1.210	-0.4777	3.00e-4	0.0013
IL-4	51	10.630	8.120	-0.3886	3.00e-4	0.0014
IL-12p70	51	0.9100	0.6500	-0.4854	7.00e-4	0.0026
IP-10	51	3.630	2.040	-0.8314	8.00e-4	0.0026
MCP-1	51	5.335	3.690	-0.5319	8.00e-4	0.0026
IL-15	50	40.130	17.355	-1.209	0.0012	0.0037
SDF-1-alpha	45	49.000	32.150	-0.6080	0.0015	0.0043
GM-CSF	51	65.430	39.390	-0.7321	0.0020	0.0052
IL-5	51	17.910	10.275	-0.8016	0.0137	0.0342
IL-18	45	10.595	6.350	-0.7386	0.0184	0.0436
HGF	51	12.880	8.440	-0.6098	0.0201	0.0452
IL-2	50	26.200	26.020	-0.0099	0.0261	0.0559
IFN-gamma	51	6.370	4.295	-0.5686	0.0341	0.0698

EGF	49	15.020	10.790	-0.4772	0.0368	0.0720
SCF	51	1.690	1.390	-0.2819	0.0512	0.0960
IL-13	51	15.620	12.165	-0.3607	0.0704	0.1268
IFN-alpha	51	0.4200	0.4950	0.2370	0.0854	0.1479
TNF-beta	50	18.788	9.250	-1.022	0.1139	0.1899
MIP-1-alpha	43	4.000	0.7000	-2.515	0.1215	0.1953
VEGF-A	51	17.610	12.710	-0.4704	0.1463	0.2269
NGF-beta	51	3.000	3.530	0.2347	0.1832	0.2748
PIGF-1	51	2.010	2.075	0.0459	0.2142	0.3018
MIP-1-beta	49	4.950	4.550	-0.1216	0.2146	0.3018
IL-7	51	0.6200	0.5800	-0.0962	0.2831	0.3861
IL-1-beta	51	3.390	3.320	-0.0301	0.3682	0.4873
IL-31	38	24.980	22.235	-0.1679	0.4046	0.5198
IL-6	38	64.040	42.670	-0.5858	0.4210	0.5198
IL-1RA	23	60.535	62.295	0.0413	0.4274	0.5198
LIF	51	6.270	6.270	0.0000	0.4590	0.5435
IL-9	51	11.640	7.150	-0.7031	0.5738	0.6621
IL-21	41	6.490	5.390	-0.2679	0.7216	0.8118
FGF-2	43	5.620	5.390	-0.0603	0.7605	0.8347
IL-1-alpha	41	0.1950	0.2650	0.4425	0.8666	0.9140
IL-17A	51	10.420	8.785	-0.2462	0.8734	0.9140
IL-8	51	4.140	1.625	-1.349	0.9500	0.9716
IL-10	51	0.8000	0.8800	0.1375	0.9963	0.9963

20 of 45 analytes showed statistically significant decreases from the acute phase to 12 months at FDR < 0.05; no analyte showed a significant increase.

Figure legends

Supplementary Figure S1. *Plausible causal structures underlying the cross-sectional HGF_{12m}–Δ(T/S) association.*

Three alternative directed acyclic graphs (DAGs) that are equally consistent with the observed inverse correlation between residual hepatocyte growth factor (HGF) at 12 months and the longitudinal change in leukocyte telomere length (Δ(T/S) = $T/S_1 - T/S_0$), but which cannot be discriminated from each other in the present observational design. ***(A)*** *HGF as upstream driver of telomere stress: elevated residual circulating HGF, as a component of the senescence-associated secretory phenotype (SASP), promotes DNA-damage signaling at telomeres and accelerates telomere shortening.* ***(B)*** *Reverse causation: pre-existing senescent or short-telomere cells secrete higher levels of HGF (SASP), such that HGF measured at 12 months is a downstream marker of cells already committed to senescence rather than a cause of telomere shortening.* ***(C)*** *Unmeasured confounding: an exposure not captured in the present protocol — for example, a residual viral reservoir, persistent low-grade systemic inflammation, or BMI/lifestyle factors — drives both elevated residual HGF and altered telomere dynamics independently; the observed HGF–Δ(T/S) correlation (dashed grey arrow) is therefore non-causal. Solid arrows represent hypothesized causal directions; the dashed grey arrow in panel (C) marks the observed statistical association in the absence of a direct causal link. The present cross-sectional observational design adjudicates none of these structures; their formal discrimination requires controlled longitudinal designs with pre-infection LTL measurements, orthogonal LTL methodologies, and assessment of candidate confounders.*
